# 17. A Retrospective Cohort Study of Influenza Infected Multiple Myeloma Patients Comparing Clinical Outcomes Between Vaccinated and Unvaccinated

**DOI:** 10.1093/ofid/ofab466.219

**Published:** 2021-12-04

**Authors:** Taylor D Wilson, Jacob Leffert, Juan Carlos Rico Crescencio, Mitchell Jenkins, Mary J Burgess

**Affiliations:** 1 University of Arkansas for Medical Sciences, Trumann, Arkansas; 2 Conway Regional Hospital, Conway, Arkansas

## Abstract

**Background:**

The current standard of care for multiple myeloma (MM) patients is to administer the influenza vaccine (InfV) annually. While in immunocompetent patients, the influenza vaccine is associated with significant benefit in morbidity and mortality, the inherent immunodeficiency from MM and its treatments reduce the InfV efficacy but it is thought to have some benefit. The effect on morbidity and mortality in MM patients has not been evaluated. Our study aims to investigate whether InfV vaccination status affects outcomes of MM patients diagnosed with Influenza A or B (FluA, FluB).

**Methods:**

This was a retrospective study, using Arkansas Clinical Data Repository, which identified all MM patients diagnosed with FluA or FluB during five consecutive flu seasons from September 1^st^ to April 30^th^, 2015-2020. Those with hospital-acquired influenza were excluded. The outcome data were collected for 30 days following the initial diagnosis. Fisher Exact test was used to compare categorical variables, and Mann Whitney U test to compare continuous variables.

**Results:**

We identified 194 MM patients diagnosed with FluA or FluB. Sixty-five (34%) were vaccinated and 129 (66%) were not vaccinated. A total of 87 (45%) were admitted to the hospital. Twenty-five (38%) of the vaccinated vs. 62 (48%) of the unvaccinated group were hospitalized (p=0.22), and 4/65 vaccinated vs. 12/129 unvaccinated required ICU treatment (p=0.59). Two patients in the vaccinated and 3 in the non-vaccinated group were intubated (p=1). The mean length of stay (LOS) for the vaccinated and unvaccinated was 10 days and 14 days, respectively, which was not significantly different (p=0.197). Two (3%) patients died within 30 days of diagnosis in the vaccinated group while four (3%) died in the unvaccinated group (p=1).

**Conclusion:**

The InfV status of MM patients had no effect on outcomes including the need for hospital admission, ICU stay, mechanical ventilation, LOS, and death. Hospitalization was common, but severe illness requiring ICU care and intubation were less common. Six patients died within 30 days of influenza diagnosis. Vaccination strategy, including high-dose and repeat doses, should be examined in MM patients.

**Disclosures:**

**All Authors**: No reported disclosures

